# A new crystal form of GABARAPL2

**DOI:** 10.1107/S2053230X21004489

**Published:** 2021-04-30

**Authors:** Kristen Scicluna, Grant Dewson, Peter E. Czabotar, Richard W. Birkinshaw

**Affiliations:** a Walter and Eliza Hall Institute of Medical Research, 1G Royal Parade, Parkville, VIC 3052, Australia; b Department of Medical Biology, The University of Melbourne, Melbourne, VIC 3052, Australia

**Keywords:** GABARAPL2, GATE-16, Atg8, autophagy, autophagosome, LIR, LC3

## Abstract

The Atg8 protein family comprises the GABA type A receptor-associated proteins (GABARAPs) and microtubule-associated protein 1 light chains 3 (MAP1LC3s) that are essential mediators of autophagy. The LC3-interacting region (LIR) motifs of autophagy receptors and adaptors bind Atg8 proteins to promote autophagosome formation, cargo recruitment, and autophagosome closure and fusion to lysosomes. A crystal structure of human GABARAPL2 has been published [PDB entry 4co7; Ma *et al.* (2015), *Biochemistry*, **54**, 5469–5479]. This was crystallized in space group *P*2_1_ with a monoclinic angle of 90° and shows a pseudomerohedral twinning pathology. This article reports a new, untwinned GABARAPL2 crystal form, also in space group *P*2_1_, but with a 98° monoclinic angle. No major conformational differences were observed between the structures. In the structure described here, the C-terminal Phe117 binds into the LIR docking site (LDS) of a neighbouring molecule within the asymmetric unit, as observed in the previously reported structure. This crystal contact blocks the LDS for co-crystallization with ligands. Phe117 of GABARAPL2 is normally removed during biological processing by Atg4 family proteases. These data indicate that to establish interactions with the LIR, Phe117 should be removed to eliminate the crystal contact and liberate the LDS for co-crystallization with LIR peptides.

## Abstract 2

A new untwinned crystal form of the autophagy protein GABARAPL2 is described. The LC3-interacting region (LIR) docking site is blocked by a crystal contact with the C-terminal residue, Phe117. This residue is removed during the processing of GABARAPL2 by Atg4 family proteases. This suggests that the removal of Phe117 is required to co-crystallize LIR peptides with GABARAPL2 in order to establish the interactions between these important autophagy mediators.

## 1. Introduction   

Autophagy is a fundamental cellular degradation pathway that is required for the homeostatic recycling of cellular components and organelles (Mizushima, 2007 ▸). Dysregulation of the autophagy pathway promotes cancer progression, neuro­degeneration, immune disorders and ageing (Dikic & Elazar, 2018 ▸). A key step in autophagy is the formation of the isolation membrane, a double-membrane structure which upon sealing forms autophagosomes that fuse with lysosomes to degrade their contents. The Atg8 proteins play a vital role in the expansion, recruitment of specific cargo by and sealing of the isolation membrane (Nguyen *et al.*, 2016 ▸; Weidberg *et al.*, 2010 ▸). In mammals, there are six Atg8 orthologs divided into two subcategories: the LC3 and GABARAP subfamilies (He *et al.*, 2003 ▸; Xin *et al.*, 2001 ▸). Although the specific roles and functional divergence of the family members are poorly understood, each undergoes post-translational processing by Atg4 family cysteine proteases. Atg4 proteins cleave Atg8 proteins at their C-termini, exposing a conserved glycine that can then be conjugated with a membrane-resident phosphatidylethanolamine (PE) lipid (Kabeya *et al.*, 2004 ▸). Once anchored, Atg8 proteins recognize autophagy receptors and adaptors by their canonical LIR motif, a small motif comprising Θ_0_-*X*_1_-*X*_2_-Γ_3_, where Θ represents an aromatic residue (W/F/Y), Γ an aliphatic residue (L/V/I) and *X* any amino acid (Noda *et al.*, 2010 ▸; Birgisdottir *et al.*, 2013 ▸). In selective autophagy, Atg8 proteins on the inner autophagosome membrane bind the LIR motifs of autophagy ‘receptors’, facilitating the encapsulation of a specific cargo in the nascent autophagosome. Additionally, Atg8 proteins can also bind the LIR motifs of autophagy ‘adaptors’, which have functions beyond degradation, including autophagosome formation, transport and fusion with lysosomes (Wirth *et al.*, 2019 ▸). Understanding the structural basis for the interaction of Atg8 proteins with specific LIR motifs is crucial for elucidating what it is that drives the specificity of the Atg8 family (Kirkin & Rogov, 2019 ▸).

GABARAPL2 (also called GATE-16) belongs to the GABARAP subfamily of Atg8 proteins. Like other Atg8 proteins, GABARAPL2 is comprised of an N-terminal helical extension preceding four β-sheets in a ubiquitin-like β-grasp fold (Ma *et al.*, 2015 ▸). This fold forms the LIR docking site (LDS), consisting of hydrophobic pocket 1 (HP1) and hydrophobic pocket 2 (HP2). The aromatic 0 and aliphatic +3 residues of LIR motifs insert into HP1 and HP2, respectively. In this work, we solved the apo structure of GABARAPL2 to 1.9 Å resolution in a new crystal form in an attempt to co-crystallize it with an LIR peptide (Otsu *et al.*, 2015 ▸). The structure reported here shares the same *P*2_1_ space group as the published structure, but showed a different monoclinic angle and is not twinned. In these structures, the C-terminal Phe117 binds in the HP1 pocket of a neighbouring molecule, thereby blocking the LDS and providing a rationale for the absence of bound LIR peptide.

## 2. Materials and methods   

### 2.1. Expression and purification of GABARAPL2   

GABARAPL2 was expressed and purified as described previously (Muhlinen *et al.*, 2012 ▸). Briefly, *Escherichia coli* strain BL21 (DE3) cells were transformed with a pETM-30 plasmid harbouring the GABARAPL2 gene (Table 1 ▸). The cells were grown in 1 l Luria Broth (LB) medium supplemented with 100 mg l^−1^ kanamycin at 37°C with agitation. Gene expression was induced when the cell density (OD_600_) reached approximately 0.6 by the addition of 500 m*M* isopropyl β-d-1-thiogalactopyranoside (IPTG) for 3 h. Cell pellets were obtained by centrifugation at 5000*g* for 10 min and were lysed by sonication in lysis buffer (50 m*M* Tris pH 8.0, 150 m*M* NaCl, 1 m*M* EDTA) at 4°C. The lysate was clarified by centrifugation at 45 000*g* for 45 min and was filtered through a 45 µm filter and immobilized on a column containing 1 ml Gluthathione Sepharose 4B resin (GE Life Sciences). GABARAPL2 was cleaved from the GST fusion protein on-column with 200 µg TEV protease and 0.5 m*M* TCEP overnight at 4°C. The eluate was concentrated to a final volume of 500 µl at 4000*g* in a 10 kDa cutoff Amicon Ultra-15 concentrator (Millipore) and was loaded onto a Superdex S75 10/100 GL gel-filtration column (GE Healthcare) equilibrated with TBS (20 m*M* Tris pH 8.0, 150 m*M* NaCl). Fractions containing cleaved GABARAPL2 were pooled and concentrated to a final concentration of 6.17 mg ml^−1^ at 4000*g* in a 10 kDa cutoff Amicon Ultra-4 concentrator (Millipore) for use in crystallization experiments.

### 2.2. Surface plasmon resonance   

Surface plasmon resonance (SPR) binding assays were performed using a BIAcore S200 in SPR buffer consisting of 10 m*M* HEPES, 150 m*M* NaCl, 3.4 m*M* EDTA, 0.05% Tween pH 7.4. NDP52 I133W LIR peptide (ENEEDWLVVTTQGE; Mimotopes) was captured by an N-terminal LC-biotin tag to the surface of a streptavidin sensor chip (GE Healthcare). The protein was reconstituted in SPR buffer in a threefold, ten-step dilution series and injected over the chip for 160 s with an 800 s dissociation time. The sensor surface was regenerated with SPR buffer supplemented with 0.1% SDS for 60 s, followed by 100 m*M* HCl for 60 s and then 50 m*M* glycine pH 2.1 for 60 s between each cycle before repeating the sample injections. The sensorgrams were double referenced by subtracting a blank SPR buffer-only sample and using a biotin-blocked reference flowcell. The sensorgrams were analysed at steady state using a report point 145 s after injection, averaging the response over 5 s. The curves were fitted using a steady-state binding model and a dose–response curve was fitted to derive the dissociation constant (*K*_d_).

### 2.3. Crystallization   

Crystallization trials were performed with purified GABARAPL2 mixed in a 1:3 ratio with a 12-mer LIR peptide with an N-terminal LC-biotin tag. The GABARAPL2–LIR peptide sample was set up in 96-well sitting-drop plates with a drop size of 300 µl at 20°C at the CSIRO Collaborative Crystallisation Centre facility. Crystals formed after seven days in several conditions from the Shotgun and Index screens exclusively containing 25–30%(*w*/*v*) polyethylene glycol (PEG) 2000/3350/4000 or PEG monomethyl ether 2000 (Table 2 ▸). The crystals were cryoprotected in well solution supplemented with 15% ethylene glycol prior to flash-cooling in liquid nitrogen.

### 2.4. Data collection and processing   

Data sets were collected at 100 K on the MX1 beamline at the Australian Synchrotron at a wavelength of 0.9637 Å using an EIGER 9M detector (Cowieson *et al.*, 2015 ▸). Crystals which formed in 0.15 *M* KBr, 30%(*w*/*v*) polyethylene glycol monomethyl ether diffracted to 1.9 Å resolution and the data were indexed, integrated and scaled using the Australian Synchrotron autoprocessing software with *XDSME* (Legrand, 2017 ▸), a Python wrapper that utilizes *XDS* (Kabsch, 2010 ▸), *POINTLESS* (Evans, 2006 ▸) and *AIMLESS* (Evans & Murshudov, 2013 ▸) (Table 3 ▸). Data quality was assessed by *phenix.xtriage* (Liebschner *et al.*, 2019 ▸).

### 2.5. Structure solution and refinement   

The phase problem was solved in *Phaser* (McCoy *et al.*, 2007 ▸) via molecular replacement using the coordinates of the apo GABARAPL2 structure (Ma *et al.*, 2015 ▸; PDB entry 4co7; Table 4 ▸). The structure was iteratively refined after rounds of real-space model building in *Coot* (Emsley *et al.*, 2010 ▸) and refinement with *Phenix* (Liebschner *et al.*, 2019 ▸). Noncrystallographic symmetry restraints and translation–libration–screw (TLS) groups were integrated in later stages of refinement and were determined automatically using the default parameters in the *Phenix* refinement software.

## 3. Results and discussion   

**Figure 1:**
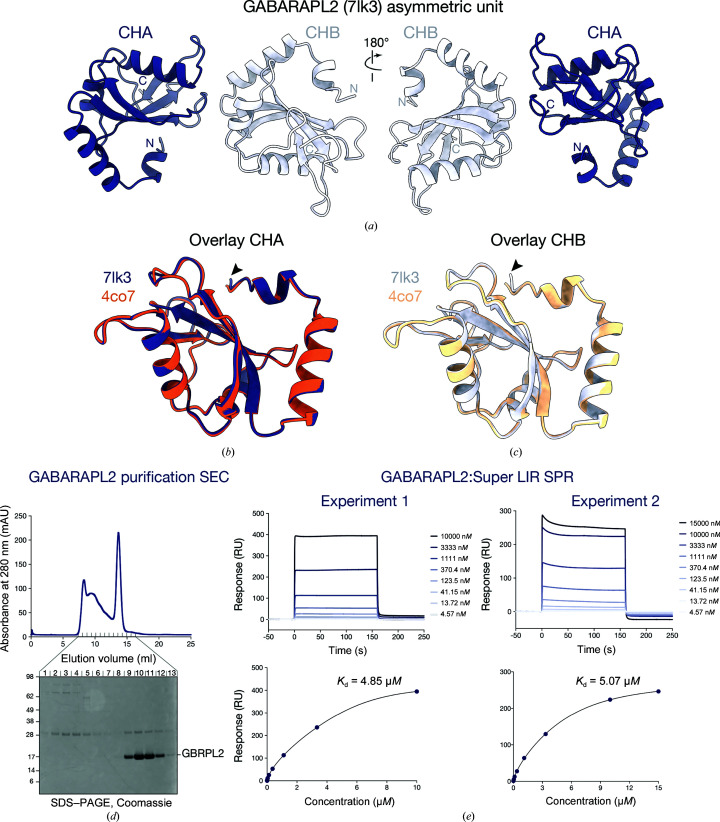
Structure and purification of full-length GABARAPL2. (*a*) The new crystal form of GABRAPL2 (PDB entry 7lk3) containing two molecules in the asymmetric unit. Chain *A* (CHA) is depicted in deep purple and chain *B* (CHB) in lilac. Overlay of (*b*) CHA from PDB entry 7lk3 with the equivalent chain of PDB entry 4co7 (orange) and (*c*) overlay of CHB from PDB entry 7lk3 (lilac) with CHB from PDB entry 4co7 (light orange). Regions of divergence in the protein backbone involving cleavage-artefact serine residues at the N-termini are shown (black arrows). (*d*) Size-exclusion chromatogram (SEC) of monomeric GABARAPL2 (GBRPL2) and SDS–PAGE gel of fractions stained with Coomassie Brilliant Blue. (*e*) A representative surface plasmon resonance sensorgram (top panel) and dose–response curve (bottom panel) for two independent experiments showing the binding of purified full-length GABARAPL2 by an NDP52 I133W LIR peptide (Super LIR) with *K*_d_ values of 4.85 and 5.07 µ*M*.

The GABARAPL2 expression and purification procedures produced 150 µl of 6.17 mg ml^−1^ monomeric GABARAPL2 largely free from GST and TEV protease contamination (Fig. 1 ▸ *d*). To confirm that the GABARAPL2 protein was functional in solution, surface plasmon resonance was used to demonstrate binding to a generic LIR peptide previously reported to engage all six Atg8 orthologs (Fig. 1 ▸ *e*; Muhlinen *et al.*, 2012 ▸). Initial screening experiments performed with the peptide in a 1:3 molar ratio produced several crystals of various sizes and quality based on visual inspection. Crystals formed in conditions containing medium-range molecular-weight PEGs from the Index and Shotgun (Fazio *et al.*, 2014 ▸) screens. We collected diffraction data from 13 crystals that diffracted to resolutions within the range 2–3 Å, with the best diffracting crystal, with dimensions of approximately 5 × 50 × 400 µm, diffracting to 1.9 Å resolution. All data sets belonged to space group *P*2_1_ with similar unit-cell dimensions, and the best diffracting crystal, which we discuss here, had unit-cell dimensions *a* = 28.4, *b* = 58.7, *c* = 69.0 Å and a monoclinic angle of 98.3° (Table 3 ▸). Intensity statistics indicated the data set was not twinned (Table 4 ▸).

Phases were obtained by molecular replacement (*Phaser-MR*) using the apo GABARAPL2 structure (PDB entry 4co7; Ma *et al.*, 2015 ▸). This published structure also crystallized in space group *P*2_1_, with unit-cell dimensions *a* = 28.7, *b* = 67.4, *c* = 58.7 Å and a monoclinic angle of 90°, producing a pseudo-orthorhombic cell that was twinned and was consequently refined in space group *P*2_1_. Despite sharing similar unit-cell dimensions, with the *b* and *c* axes permuted, the structure that we present does not show a twinned crystal pathology, which is likely to be due to the change in the monoclinic angle.

The final model (Table 5 ▸) contained two copies of the molecule in the asymmetric unit (Fig. 1 ▸ *a*) and had a solvent content of 40%, as was seen for the published GABARAPL2 structure. No gross structural differences were observed in the geometry of the protein backbone when overlaying each of the chains with their published counterparts, excluding the non-wild-type N-terminal serine residues derived from protease digestion (Figs. 1 ▸ *b* and 1 ▸ *c*; the r.m.s.d. on C^α^ atoms between chain *A* of PDB entry 4co7 and chain *A* of PDB entry 7lk3 is 0.27 Å).

**Figure 2:**
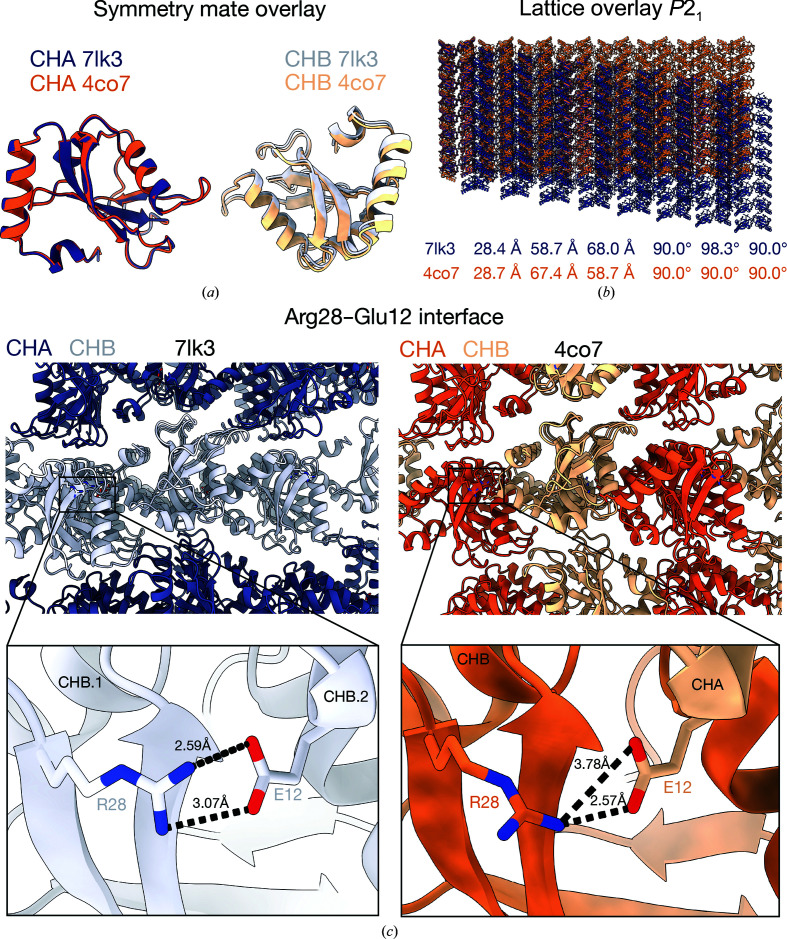
Differences in unit-cell dimensions between PDB entry 4co7 and the new crystal form of GABARAPL2 manifest in the *P*2_1_ lattices due to a unique crystal contact. (*a*) An overlay of symmetry mates between PDB entry 4co7 (orange) and the new structure (purple) after performing a *y* + 1/2, −*z* symmetry operation to align the structures with the same origin reveals a slight offset in the positioning of CHB in the published structure. (*b*) Overlay of the crystal lattices of the new crystal form (purple) and PDB entry 4co7 (orange) in space group *P*2_1_ exhibiting how the CHB offset manifests in the crystal form. Unit-cell dimensions are listed below. (*c*) A crystal contact between Arg28 and Glu12, showing the salt bridge that is bidentate in the untwinned PDB entry 7lk3 and monodentate in the published, twinned PDB entry 4co7 structure (see inlays for comparison). The interface occurs between two chains of the same type in PDB entry 7lk3 (lilac) and between chains *A* (orange) and *B* (light orange) in PDB entry 4co7.

The published GABARAPL2 structure (PDB entry 4co7) and the structure that we present here show similar unit cells with the *b* and *c* axes permuted and a differing monoclinic angle (Ma *et al.*, 2015 ▸). The two structures showed similar crystal contacts between neighbouring molecules in space group *P*2_1_. The PDB entry 4co7 structure is pseudo-ortho­rhombic due to pseudo-merohedral twinning, with a monoclinic angle of 90°. The PDB entry 4co7 structure may be described with the monoclinic angle set on either the *b* axis (as is the case in the deposited structure) or the *c* axis. This setting changes the relationship between noncrystallographic and crystallographic symmetry in the description of the crystal in PDB entry 4co7 relative to PDB entry 7lk3. Unfortunately, this changes the description of the asymmetric unit between the two structures, despite the two crystal forms sharing similar crystal packing. The crystal contact between asymmetric monomers in PDB entry 4co7 is the same as a −*x*, *y* + 1/2, −*z* (2_1_) symmetry operation in the structure that we present. In hindsight, comparison of the structures would have been simpler had the deposited PDB entry 4co7 structure been described with the monoclinic angle on the *c* axis and permuted to the *b* axis. The two crystal lattices were aligned using chain *A* and the −*x*, *y* +1/2, −*z* symmetry operation of chain *A* from the structure presented here as the aligning unit with chains *A* and *B* from the asymmetric unit of the published structure. This showed a slight offset in the overlay of chain *B* of the published structure with the equivalent molecule from the new structure (Fig. 2 ▸ *a*). This subtle change accumulates over the crystal lattice, where differences between the 98° monoclinic angle of this new structure and the 90° angle of the published structure were clearly appreciable (Fig. 2 ▸ *b*). It is not entirely clear what causes the change in the monoclinic angle. In the new crystal, there is a change in the orientation of Arg28 that allows the formation of a bidentate salt bridge to Glu12 from its +1/2, −*z* symmetry mate. This interface is monodentate in the published structure and occurs between monomers within the asymmetric unit (Fig. 2 ▸ *c*). However, it is unclear whether this change in salt-bridge orientation causes the deviation in the monoclinic angle or is a consequence. The untwinned structure used a crystallization condition consisting of 150 m*M* KBr and 30%(*w*/*v*) PEG monomethyl ether 2000 precipitant at pH 8 (no buffer was included in the crystallization condition), while the twinned structure used 100 m*M* phosphate buffer at pH 7 and 50 m*M* KCl. The divergence of these conditions makes the similarities between the structures even more striking and suggests dominance of these crystal contacts.

**Figure 3:**
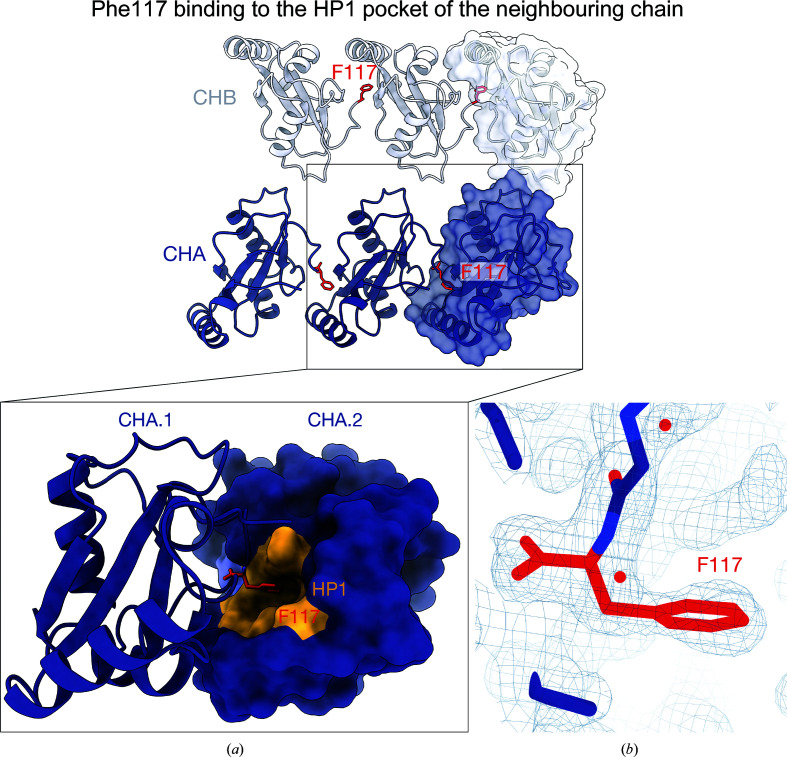
A crystal contact in the new form of GABARAPL2 occludes the LIR docking site. (*a*) Arrangement of the new GABARAPL2 crystal form lattice showing Phe117 (red) binding to the HP1 pocket (yellow) of a neighbouring molecule of the same chain type. (*b*) The 2*F*_o_ − *F*_c_ electron-density map is shown at 1σ and illustrates the density of Phe117 (red).

**Figure 4:**
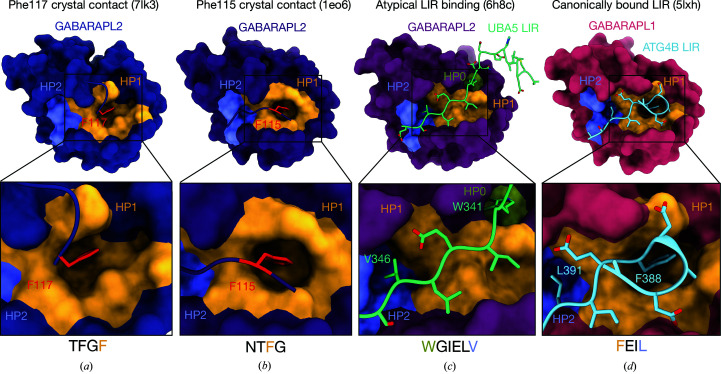
Comparison of phenylalanine-directed crystal contacts between HP1 of neighbouring molecules with canonical and noncanonical LIR peptides. (*a*) The interface between Phe117 (red) of GABARAPL2 (deep purple) and the HP1 pocket (yellow) of a neighbouring molecule in PDB entry 7lk3 and (*b*) the interface of an alternative crystal contact that can form between Phe115 (red) of GABARAPL2 (purple) and HP1 of a neighbouring molecule (PDB entry 1eo6). (*c*) The only structure of GABARAPL2 (magenta) bound to a LIR peptide (green) is noncanonical and binds in an atypical manner (PDB entry 6h8c). The UBA5 LIR peptide contains two extra residues within the core LIR, flanked by the typical aromatic residue at position 0 (Trp341), which binds to a novel HP0 pocket (olive). However, the aliphatic residue (Val346) that typically occurs at the +3 position in canonical LIR motifs is in the +6 position and binds to the HP2 pocket (purple), modifying the topology of the HP1 pocket. (*d*) GABARAPL1 (pink) bound to the Atg4B LIR (blue) (PDB entry 5lxh; Skytte Rasmussen *et al.*, 2017 ▸) provides an example of how a canonical LIR peptide binds to the Atg8 HP1 pocket and adopts a similar conformation to the GABARAPL2 Phe117–HP1 crystal contact seen in PDB entry 7lk3. PDB entry 5lxh was used as an example as there are no GABARAPL2 structures bound to canonical LIR peptides available in the PDB, and the 0 residue of the Atg4B LIR peptide is a phenylalanine, as per the interface of PDB entry 7lk3. This Phe388 residue inserts into the HP1 pocket (yellow) of GABARAPL1, and Leu391 at the +3 position inserts into the HP2 pocket (light purple).

The crystal contact with the most extensive buried surface area in the structure occurs between the C-terminal Phe117 and HP1 of a neighbouring symmetry molecule, with a buried surface area of ∼550 Å^2^ (Figs. 3 ▸ *a*, 3 ▸ *b*, 3 ▸ *c* and 4 ▸ *a*). This is observed and described in the published structure with PDB code 4co7. In addition to PDB entry 4co7, another crystal structure of GABARAPL2, PDB entry 1eo6 (Paz *et al.*, 2000 ▸), has been reported. This is annotated in the PDB as bovine in origin; however, the human and bovine GABARAPL2 sequences are identical. In this structure (PDB entry 1eo6) there are two molecules in the asymmetric unit. In one molecule Phe117 also interacts with HP1 of the neighbouring molecule in the crystal, as observed in PDB entries 4co7 and 7lk3. However, there is an altered interaction for the second molecule, where Phe115 inserts into HP1 of the neighbouring molecule (Fig. 4 ▸ *b*). We note that the Atg8 family HP1 serves as the docking site for the aromatic Θ_0_ residue of an LIR motif (Figs. 3 ▸ *b* and 4 ▸). The occlusion of HP1 by Phe117 from the crystal contact is likely to account for the absence of bound LIR peptide in the crystals (Fig. 4 ▸). Whilst the full-length GABARAPL2 construct can bind the LIR peptide in SPR experiments (Fig. 1 ▸ *e*), demonstrating that the HP1 site is unoccupied in solution, attempts to co-crystallize the LIR peptide with Phe117-deficient GABARAPL2 in the same crystallization screens were unsuccessful, providing additional support for this residue mediating crystallization in the apo form. Our ongoing investigation of GABARAPL2 will use this Phe117-truncated construct to obtain complexes with LIR peptides.

This also provides a potential explanation for the absence of published crystal structures of GABARAPL2 bound to canonical LIR peptides, as this crystal form requires a contact between the C-terminal Phe117 and the LIR-binding site of a symmetry molecule. To date, there is a single structure of human GABARAPL2 bound to a LIR peptide with a C-terminal Phe117 truncation (PDB entry 6h8c; Huber *et al.*, 2020 ▸). However, this structure was solved by NMR and contains an atypical LIR motif comprising six amino acids, with Trp341 at the 0 position binding to a novel HP0 pocket and Val346 at the +6 position binding to HP2 (Fig. 4 ▸ *c*). This is an alternative binding mechanism and is not representative of how a canonical LIR motif is thought to bind to GABARAPL2 based on comparison with GABARAPL1 structures with LIR peptides (Fig. 4 ▸ *d*). Intriguingly, in mammalian Atg8 family proteins all residues C-terminal to Gly116 (GABARAPL2 numbering) are cleaved by Atg4 family proteases in the initial processing steps of autophagosome formation and PE conjugation. This provides a rationale to remove C-terminal Atg8 protein residues preceding the glycine that becomes exposed after Atg4 cleavage. Phe117 of GABARAPL2 is poorly conserved across Atg8 orthologs. Phe117 is only conserved in mammalian LC3A and is absent in all other eukaryotic Atg8 family members (Schaaf *et al.*, 2016 ▸). As Phe117 of GABARAPL2 forms a dominant crystal contact that blocks LIR peptide binding, this suggests that future co-crystallization studies should remove this residue to enable new crystal forms that permit LIR peptide binding. Co-crystallization of GABARAPL2 with various LIR peptides will produce new insights into the specificity of these interactions and enable the deconvolution of the diverse range of bio­logical functions shown by Atg8 family members.

## Supplementary Material

PDB reference: untwinned human GABARAPL2, 7lk3

## Figures and Tables

**Table 1:** Macromolecule-production information

Source organism	*Homo sapiens*
DNA source	Synthetic DNA
Expression vector	pETM-30
Expression host	*E. coli* BL21 (DE3)
Complete amino-acid sequence of the construct produced	GAMSMKWMFKEDHSLEHRCVESAKIRAKYPDRVPVIVEKVSGSQIVDIDKRKYLVPSDITVAQFMWIIRKRIQLPSEKAIFLFVDKTVPQSSLTMGQLYEKEKDEDGFLYVAYSGENTFGF

**Table 2:** Crystallization

Method	Sitting-drop vapour diffusion
Plate type	Innovaplate SD-2 96-well
Temperature (K)	294
Protein concentration (mg ml^−1^)	6.17
Buffer composition of protein solution	20 m*M* Tris pH 8.0, 150 m*M* NaCl (TBS)
Composition of reservoir solution	0.15 *M* KBr, 30%(*w*/*v*) PEG monomethyl ether 2000
Volume and ratio of drop	0.15 µl protein solution:0.15 µl reservoir solution
Volume of reservoir (µl)	50

**Table 3:** Data collection and processing

Diffraction source	MX1, Australian Synchrotron
Wavelength (Å)	0.9537
Temperature (K)	100
Detector	EIGER 9M
Crystal-to-detector distance	210
Rotation range per image (°)	1
Total rotation range (°)	360
Exposure time per image (s)	18
Space group	*P*2_1_
*a*, *b*, *c* (Å)	28.4, 58.7, 68.0
α, β, γ (°)	90.0, 98.3, 90.0
Resolution range (Å)	33.65–1.90 (1.968–1.900)
Total reflections	122554 (12251)
Unique reflections	17461 (1693)
Completeness (%)	99.58 (99.94)
Multiplicity	7.0 (7.2)
Mean *I*/σ(*I*)	14.55 (2.00)
*R*_merge_	0.09293 (0.9177)
CC_1/2_	0.999 (0.806)
Average *B* factor (Å^2^)	25.38

**Table 4:** Twinning and intensity statistics

〈*I* ^2^〉/〈*I*〉^2^	2.157 (untwinned, 2.0; perfect twin, 1.5)
〈*F*〉^2^/〈*F* ^2^〉	0.774 (untwinned, 0.785; perfect twin, 0.885)
〈|*E* ^2^ − 1|〉	0.770 (untwinned, 0.736; perfect twin, 0.541)
〈|*L*|〉, 〈*L* ^2^〉	0.500, 0.331
Multivariate *Z*-score *L*-test	1.864

**Table 5:** Structure refinement

Resolution range (Å)	33.65–1.90 (1.968–1.900)
Molecules in the asymetric unit	2
Reflections used in refinement	17445 (1693)
Reflections used for *R*_free_	1761 (180)
*R*_work_	0.1958 (0.2864)
*R*_free_	0.2326 (0.3351)
R.m.s. deviations	
Bond lengths (Å)	0.007
Angles (°)	0.90
Ramachandran plot	
Favoured regions (%)	99.14
*MolProbity* score	1.04
